# Community pharmacists’ knowledge, practices and beliefs about complementary and alternative medicine in Palestine: a cross-sectional study

**DOI:** 10.1186/s12906-017-1940-8

**Published:** 2017-08-29

**Authors:** Naser Y. Shraim, Ramzi Shawahna, Muna A. Sorady, Banan M. Aiesh, Ghadeer Sh. Alashqar, Raghad I. Jitan, Waed M. Abu Hanieh, Yasmeen B. Hotari, Waleed M. Sweileh, Sa’ed H. Zyoud

**Affiliations:** 1 Department of Pharmacy, College of Medicine and Health Sciences, An-Najah National University, Nablus, Palestine; 2 Department of Pharmacology and Toxicology, College of Medicine and Health Sciences, An-Najah National University, Nablus, Palestine; 3 PharmD program, College of Medicine and Health Sciences, An-Najah National University, Nablus, Palestine; 4 Palestine Department of Clinical and Community Pharmacy, College of Medicine and Health Sciences, An-Najah National University, Nablus, Palestine

**Keywords:** CAM, Pharmacists, Knowledge, Beliefs, Practice, Information sources, Palestine

## Abstract

**Background:**

Complementary and alternative medicine (CAM) utilization is dramatically increasing among patients. As community pharmacies are a major provider of CAM products, community pharmacists need to have the sufficient knowledge and information to advice their patients, answer their inquiries and to be proactive in the healthcare process to ensure optimal therapy outputs and minimize both drug-drug and drug-herb interactions. Therefore, the main objective of this study was to assess the knowledge, beliefs, and practices of community pharmacists in Palestine about CAM.

**Methods:**

The study was conducted in a cross-sectional design in which a questionnaire was administered on a sample of licensed community pharmacists from Palestine. The questionnaire was of 5 sections: demographic and practice details of the participants, practice, beliefs, and knowledge about CAM. Mann-Whitney-*U* or Kruskal-Wallis tests were used to comparison of different issues as appropriate. *P*-values of <0.05 were considered significant.

**Results:**

A total of 284 community pharmacists were surveyed, however, 281 were included in the analysis as they met inclusion criteria. Out of the 281, 149 (53.0%) of the participants were males and the rest were females. About 40% of the participants were between 20 to 29 years old. Pharmacists frequently recommended CAM modalities. Exercises (84.0%) and food supplements (82.6%) were the most commonly recommended modalities. In the last year, vitamin B_12_ was the most frequently prescribed supplement. The median knowledge score was 5 out of 8 and the median beliefs about CAM score was 4.0 out of 7.0.

**Conclusions:**

CAM recommendations by pharmacists appear to be commonplace. Although their knowledge scores were fair to average, pharmacists still need more education and training about CAM in order to be more qualified to provide better pharmaceutical care and improve their patient’s outcome.

## Background

Complementary and alternative medicine (CAM) is commonly used in different nations around the world [1]. A complementary therapy means that it can be used alongside with conventional medical treatment, whereas, an alternative therapy is generally used instead of conventional medical treatment. Studies have shown that CAM consumers generally use different products after being recommended by their families, friends, herbalists, pharmacy assistants, pharmacists and physicians [2]. Because of patient dissatisfaction with conventional medicines and the high costs of these medications, the use of CAM products in the treatment of diseases has increased dramatically [1, 3, 4]. It is believed that socio-demographic characteristics influence patient’s intention to use CAM before or as a complement to conventional medicine. It is recommended that these factors should be considered by providers of conventional healthcare [5].

Previous studies reported that some patients are using CAM products as alternatives to conventional medicines, however, other patients are using them concomitantly with their prescribed medications [6]. As community pharmacies are major provider of CAM products, pharmacists need to have good knowledge on the appropriate way to use these products. Additionally, many CAM consumers seek advice on their CAM use from community pharmacists [1]. Pharmacists are key healthcare providers, therefore, they have a professional obligation to provide quality information and guidance to their patients including those using CAM [7]. Besides, patients generally tend to reveal their use of CAM products to their pharmacists rather than their physicians [6]. Many of CAM users believe that pharmacists should take their role in providing information pertaining the safety and effectiveness of CAM. Pharmacists should also be able to check any potential drug interactions with CAM products [7]. Although pharmacists generally agree that they should provide these information, but many of them feel that they have insufficient knowledge and/or inadequate education about CAM [8–12]. This lack of knowledge refers to many nested factors. First, pharmacy schools provide wide variety of academic plans for obtaining the pharmacy degree, in which CAM courses and topic to be covered during the syllabus are varying among different institutions. Besides, there are no institutional rules to teach CAM courses in the pharmacy curricula [13, 14]. Integrating CAM education in the pharmacy curricula can complement current pharmacy education [15]. Second, pharmacists think that there is a lack of accurate and easily accessible information, including good patient resources [8]. Third, lack of training, reimbursement, and time constraints are obstacles that prohibit pharmacists from providing optimal patient care [8, 16–19].

Little research was conducted on integrating CAM into pharmacy practice and pharmacists’ ability to meet their patients’ needs for CAM. Ultimately, when a pharmacist fail to efficiently educate CAM users on the best ways to use these products, patients might suffer [20, 21]. There is clear evidence supporting the growing interest in CAM among patients in Palestine [22]. To date, the majority of studies conducted in Palestine have focused on knowledge and attitude of diabetic, hypertensive, cancer, hemodialysis patients, and students pertaining to CAM or herbal use [23–28]. However, few studies were conducted in the Middle East region investigating knowledge, practice and beliefs about CAM among health care providers, particularly community pharmacists [6, 29–32]. Therefore, the main objective of this study was to assess the knowledge, beliefs, and practices of community pharmacists in Palestine about CAM.

## Methods

### Study design

This study was conducted in a cross-sectional design. A questionnaire was administered on licensed community pharmacists.

### Population and setting

The current study was conducted on community pharmacists practicing in the West Bank of Palestine. The total number of licensed community pharmacies in the West Bank is about 942. We intended to recruit one pharmacist from each pharmacy. According to the Palestinian Pharmaceutical Association-Jerusalem Center, the West Bank is divided into 8 governorate sub-committees. These governorates are: East-Jerusalem, Ramallah, Bethlehem, Hebron, Jenin, Nablus, Tulkarm, and Qalqilia).

### Sample size and sampling procedure

Using the number of community pharmacies in the West Bank, the sample size was estimated. Raosoft sample size calculator: (http://www.raosoft.com/samplesize.html) was used with pre-determined margin of error of 5%, and confidence level of 95%. In order to minimize erroneous results and to increase the reliability of this study, the target sample size was set to be 281 pharmacists. A convenience sampling technique was used in this study. Eligible participants had to meet a set of inclusion criteria. These criteria are: Palestinian nationality only; licensed pharmacist in Palestinian Ministry of Health; had Bachelor degree certification at least or higher degree; willing to participate and who had provided verbal consent to participate in the study; and completely filled the questionnaire form and answered all questions.

### Data collection form

The questionnaire used in the study was developed following a detailed review of the relevant literature [12, 29, 31, 33–36]. The questionnaire contained both closed and open-ended questions and it was divided into five sections. Section A consisted of questions about the socio-demographic and other background characteristics. Section B collected the practice details of the participants. Pharmacists were asked about their personal use of CAM, the reasons for using CAM, factors that affected their recommendation of CAM, degree of satisfaction with CAM, and whether they have advised their patients to use CAM within the last year. In section C, pharmacists were asked 7 questions on their beliefs about CAM. Questions were on CAM safety, adverse effects, benefits, placebo effects and pharmacists level of confidence in CAM [33, 37]. Pharmacists had to respond on a Likert scale of 5, where, 1 indicated strong disagreement and 5 indicated strong agreement. Besides, this part contained a question about the barriers that limit the appropriate use of CAM**,** and the information the pharmacist needs to know about CAM such as (drug interaction, use in pregnancy, side effects, dose, uses, and others) [37]. Section D collected participants’ knowledge. The knowledge was assessed for herbal and traditional herbs because it is the most commonly encountered type of CAM in community pharmacies in Palestine and the use of such type of CAM bears several therapeutic consequences. This section consisted of 8-item knowledge test statements on uses, adverse effects, and contraindications of CAM intended to examine the pharmacist’s knowledge. The respondent was given three options: “True”, “False”, and “I do not know”. In scoring the pharmacists’ knowledge, a score of one was given to correct answers and score of zero was used for incorrect/don’t know answers. The total scores range from 0 to 8 in which higher scores mean greater knowledge. Section E collected the resources that pharmacists used for seeking information about CAM [29]. Face and content validity of the final questionnaire was discussed and judged by a panel of three specialist pharmacists who are experts in the field of CAM for assessing the organization, clinical terminology, meaning of terms, completeness, appropriateness and logical sequence of the statements, and the accuracy. Some questions were modified as necessary. The questionnaire was piloted among a sample of 30 pharmacists at the final year of the study to test the readability and reliability. Results from the pilot testing were not included in the final analysis of the data. The survey instrument was reviewed and improvements were made based on the feedback received in the pilot. The questionnaire was finalized in Arabic language. The data were collected in the period of September 2014 to January 2015.

### Statistical analysis

Data were analyzed using Statistical Package for Social Sciences version 16 (SPSS 16). Frequencies and percentages of responses were generated for each answer in the questionnaire. Mann-Whitney-*U* test or Kruskal-Wallis test were used to compare differences as appropriate. *P*-values of <0.05 were considered statistically significant [22].

### Ethical approval

The full research protocol was approved by the Institutional Review Board (IRB) at An-Najah National University before the initiation of this study. Besides, informed verbal consent was obtained from the pharmacists prior to the initiation of the study.

## Results

### Demographic characteristics of the participant pharmacists

A total of 313 community pharmacists were asked to participate in the study, 284 pharmacists out of them accepted to fill out the questionnaire with a response rate of 90.7%. However, data from only 281 were included in the analysis. Data of 3 pharmacists were excluded because they did not meet the inclusion criteria. The demographic characteristic of the pharmacists are summarized in (Table 1). Of the 281 pharmacists, 149 (53.0%) were males and 132 (47.0%) were females. The highest percent of pharmacists (112, 39.9%) were between the ages of 20 to 29 year old, whereas few were above 60 years of age (12, 4.3%). The majority of pharmacists (262, 93.2%) had a bachelor degree in pharmacy, while the remaining had a higher pharmacy degree (19, 6.8%). The majority of pharmacists graduated from Palestinian universities (144, 51.2%), whereas (99, 35.2%) graduated from schools of pharmacy in Arab countries, while the remaining (38, 13.5%) graduated from Foreign universities. With regard to the experience, the majority (96, 34.2%) were between 1 and 5 years of experience, to a lesser extent (48, 17.1%) were between [11–15] years, and very few (15, 5.3%) were less than 1 year of experience.

**Table 1:** Demographic characteristics of the participant pharmacists

Variable	Frequency (Percent %)
Gender	
Male	149 (53.0)
Female	132 (47.0)
Age	
20–29	112 (39.9)
30–39	76 (27.0)
40–49	59 (21.0)
50–59	22 (7.8)
> 60	12 (4.3)
Education level	
Bachelor degree	262 (93.2)
Master degree	19 (6.8)
University of graduation	
Local	144 (51.2)
Regional	99 (35.2)
International	38 (13.5)
Experience (Year)	
< 1	15 (5.3)
1–5	96 (34.2)
6–10	45 (16.0)
11–15	48 (17.1)
16–20	32 (11.4)
> 20	45 (16.0)
Location of the pharmacy	
City	208 (74.0)
Village	65 (23.1)
Refugee Camp	8 (2.8)

### Pharmacists’ practice toward CAM

**Fig. 1:**
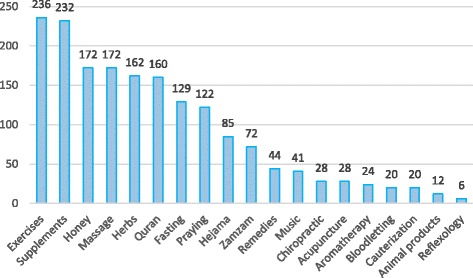
Types of CAMs recommended by pharmacists

Different types of CAMs were recommended by the Palestinian pharmacists. Exercises (84.0%) and food supplements (82.6%) were the most frequently recommended modalities. On the other hand, honey (172, 61.2%), massage (172, 61.2%) and herbs (162, 57.7%) came in the second place. (Figure 1) illustrates different types of CAM recommended by Palestinian community pharmacists.

However, ritual Islamic spirituality including recitation of Quran, fasting, praying, hejama and zamzam drinking had a reasonable percent of recommendations that can be trusted by patients as complementary therapy. Out of 281 pharmacists, 160 pharmacists (56.9%) recommended recitation of Holy Quran. One hundred and twenty nine of participant pharmacists (45.9%) believed that fasting is helpful, whereas 122 pharmacists (43.4%) considered that praying plays a crucial influence in CAM. Eighty five participants (30.2%) and 72 pharmacists (25.6%) believed that hejama and zamzam drinking respectively can be recommended. However, the least percent was for reflexology 6 (2.1%).

**Fig. 2:**
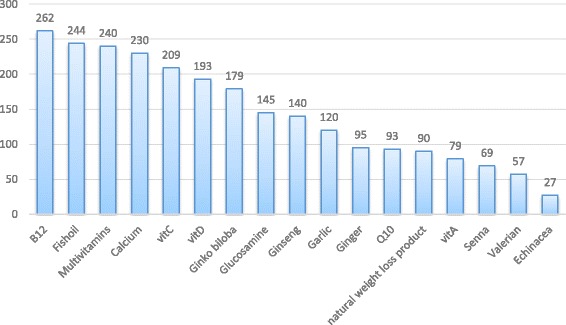
The frequencies of CAM prescribing by the pharmacists during the last year

Pharmacists were also asked whether they recommended any of the supplements and herbs shown in Fig. 2 during the last year. Vitamin B_12_ had the highest percentage such that 262 pharmacists (93.2%) recommended this essential vitamin in the last year, followed by fish oil which was recommended by 244 pharmacists (86.8%). Multivitamins and calcium were also dispensed in the last year at rate of 240 (85.4%) and 230 (81.9%) respectively. The least percent of prescribing were for valerian (*Valeriana officinalis*) and Echinacea (*Echinacea purpurea*) 57 (20.3%) and 27 (9.6%) respectively.

Table 2 illustrates factors affecting pharmacist recommendations toward specific CAM products. The majority of pharmacists reported that the two main factors affecting their recommendations are the effectiveness of the product to be scientifically proven 245 (87.2%), and the positive responses from customers on the effectiveness of the product 218 (77.6%).

**Table 2:** Motivating factors reported by the pharmacists toward recommendations for complementary and alternative medicine use

Factor	No. (%)
Product efficacy is scientifically proven	245(87.2)
Positive responses from customers on the effectiveness of the product	218(77.6)
Fewer side effects	191(68.0)
Doctors’ recommendations	114(40.6)
Less expensive (Cheaper)	100(35.6)
Publicity of the product	96(34.2)
Highest profit	56(19.9)
Recommendations from medical representative	42(14.9)
Incentives from manufacturers	39(13.9)

### Pharmacists’ beliefs about CAM

**Fig. 3:**
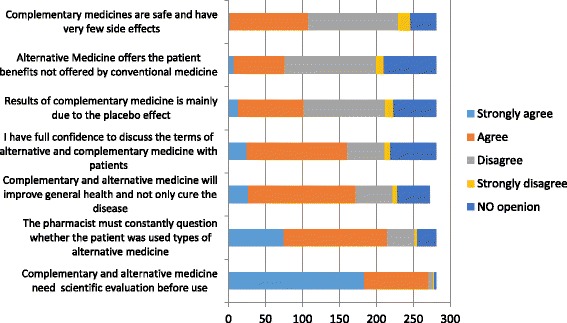
Pharmacists’ beliefs toward CAM

Figure 3 clearly shows that the majority of pharmacists 271 (96.5%) agreed that alternative medicine need scientific testing before use. Seventy six and a half percent agreed that the pharmacist should routinely question whether the patient was using any kind of alternative medicine, while 40 (14.3%) of the pharmacists disagreed. About half of the pharmacists 138 (49.1%) disagreed that herbal drugs have less side effect than conventional medicines, while 108 (38.4%) agreed. The median beliefs about CAM score value was 4.00 (interquartile range: 3–5). Table 3 summarizes our finding which revealed that there was no significant difference in beliefs about CAM score values among pharmacists. Mann-Whitney test was employed to compare pharmacists’ beliefs about CAM between male and female pharmacist. No significance differences were observed between male and female responses, with *p* values being greater than 0.05. Besides, Kruskal-Wallis test was used to examine the pharmacists; beliefs about CAM according to their age, educational level, university of graduation, experience, the city where the pharmacy located and the location of the pharmacy weather it is in city, village or camp. Similarly, no differences were detected with *p* values being greater than 0.05 in all instances.

**Table 3:** Association between sociodemographic and practice variables of the participants and median belief score

Variable	Frequency (%)	Beliefs score Median [interquartile range]	*P*-value
Gender			
Male	149 (53.0)	4.00[3.00–5.00]	0.657
Female	132 (47.0)	4.00[3.00–5.00]	
Age			
20–29	112 (39.9)	4.00[3.00–5.00]	
30–39	76 (27.0)	4.00[3.25–5.00]	
40–49	59 (21.0)	4.00[3.00–5.00]	0.242
50–59	22 (7.8)	4.00[4.00–5.00]	
> 60	12 (4.3)	4.00[3.25–4.00]	
Education level			
Bachelor degree	262 (93.2)	4.00[3.00–500]	0.722
Master degree	19 (6.8)	4.00[4.00–5.00]	
University of graduation			
Local	144 (51.2)	4.00[3.00–5.00]	
Regional	99 (35.2)	4.00[3.00–5.00]	0.765
International	38 (13.5)	4.00[4.00–5.00]	
Experience (Year)			
< 1	15 (5.3)	4.00[4.00–5.00]	
1–5	96 (34.2)	4.00[3.00–5.00]	
6–10	45 (16.0)	4.00[3.50–5.00]	0.523
11–15	48 (17.1)	4.00[4.00–5.00]	
16–20	32 (11.4)	4.00[3.00–5.00]	
> 20	45 (16.0)	4.00[4.00–5.00]	
Location of the pharmacy			
City	208 (74.0)	4.00[3.00–5.00]	
Village	65 (23.1)	4.00[3.00–5.00]	0.331
Refugee Camp	8 (2.8)	4.00[4.00–5.00]	

In addition, Pharmacists reported that the main two barriers that limit the appropriate use of CAM (Table 4) are the inadequate number of trained personnel to use CAM 232 (82.6%) and lack of scientific knowledge in CAM 207 (73.7%). However, only 9 pharmacists (3.2%) reported that there is no obstacles limiting the appropriate CAM use.

**Table 4:** The barriers that limit the appropriate use of complementary and alternative medicine

Statement	No.(%)
Small number of trained personnel to use CAM.	232 (82.6)
Lack of scientific knowledge in CAM	207 (73.7)
Lack of scientific evidence to use CAM	195 (69.4)
Lack of reliable sources of information	189 (67.3)
Need a long time of treatment	129 (45.9)
Lack of time	72 (25.6)
Lack of interest in CAM	51 (18.1)
There is no obstacle	9 (3.2)

### Pharmacists’ knowledge about CAM

The mean knowledge score values was 4.32 ± 1.78 out of 8 (mean ± SD) with a median score of 5.00 (interquartile range: 3.00–5.50). A significant difference in knowledge score value was found between pharmacists according to age, educational level, university of graduation, experience, the city where the pharmacy located and the location of the pharmacy weather it is in city, village or camp (Kruskal-Wallis test; *p*-value <0.05). Pharmacists aged between 20 to 29 had a higher knowledge score than older pharmacists. Furthermore, pharmacists with master degree scored a higher knowledge score than pharmacists with bachelor degree. The knowledge score increased in locally graduated pharmacists and in pharmacies located in villages. Unexpectedly, the study revealed that knowledge score increased as the pharmacists’ experience decreased. Tables 5 and 6 summarize our findings.

**Table 5:** Association between the sociodemographic and practice variables and median knowledge scores

Variable	Frequency (%)	Knowledge score Median [interquartile range]	*P*-value
Gender			
Male	149 (53.0)	4.00[3.00–5.00]	0.190
Female	132 (47.0)	5.00[3.00–6.00]	
Age			
20–29	112 (39.9)	5.00[4.00–6.00]	
30–39	76 (27.0)	4.50[3.00–6.00]	
40–49	59 (21.0)	4.00[2.00–5.00]	0.013
50–59	22 (7.8)	4.00[3.00–5.00]	
> 60	12 (4.3)	4.00[2.25–5.00]	
Education level			
Bachelor degree	262 (93.2)	4.00[3.00–500]	0.004
Master degree	19 (6.8)	5.00[5.00–6.00]	
University of graduation			
Local	144 (51.2)	5.00[4.00–6.00]	
Regional	99 (35.2)	4.00[3.00–5.00]	0.000
International	38 (13.5)	4.00[2.00–5.00]	
Experience (Year)			
< 1	15 (5.3)	5.00[3.00–6.00]	
1–5	96 (34.2)	5.00[4.00–6.00]	
6–10	45 (16.0)	5.00[3.00–6.00]	0.025
11–15	48 (17.1)	4.00[3.00–5.00]	
16–20	32 (11.4)	3.50[2.00–5.75]	
> 20	45 (16.0)	4.00[3.00–5.00]	
Location of the pharmacy			
City	208 (74.0)	4.00[3.00–5.00]	
Village	65 (23.1)	5.00[3.50–6.00]	0.025
Refugee Camp	8 (2.8)	4.00[1.50–5.75]

**Table 6:** Frequencies and percentages of participant pharmacists who correctly answered the 8 – knowledge questions (with correct answers provided beside each statement; T = true and F = false)

Statement	No. (%)
Senna contraindicated in case of pregnancy and children under 12 years. (T)	244 (86.8)
Eating Spinach is safe for kidney patients. (F)	188 (66.9**)**
Fenugreek increases the risk of elevated blood sugar so it should be avoided in diabetes patients. (F)	167 (59.4)
Garlic increase the possibility of bleeding when used with warfarin. (T)	163 (58.0)
Bronchoconstriction is a side effect of caffeine. (F)	156 (55.5)
Ginger is effective in decreasing PMS. (T)	146 (52.0)
Echinacea is used to suppress immunity. (F)	95 (33.8)
The use of digoxin with bran will increase the concentration of digoxin. (F)	55 (19.6)

*PMS* Premenstrual syndrome

**Fig. 4:**
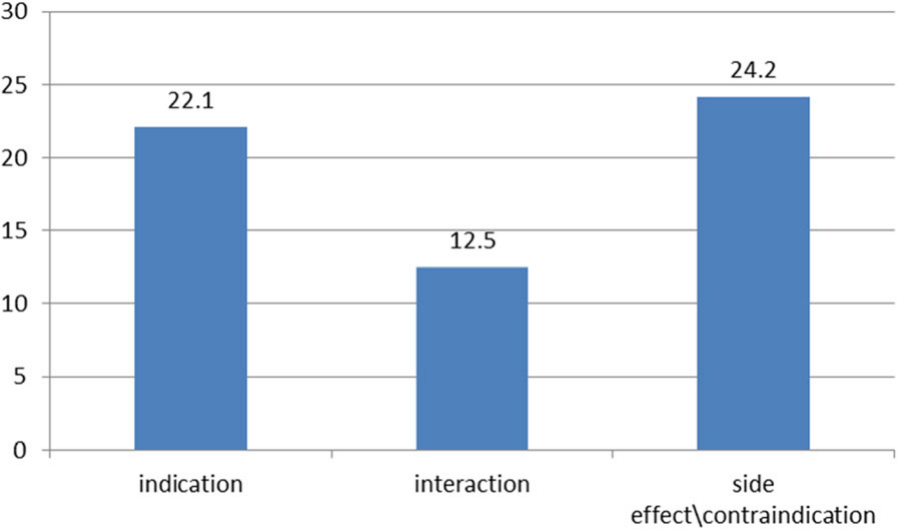
Percentage of participants who correctly answered all statements regarding various aspects in knowledge section

In addition, our results showed that 24.2% of the participant pharmacists answered correctly all statements pertaining the side effect and contraindication of the herbal products. Comparably, 22.1% of the pharmacists fulfilled the question with correct answers concerning CAM indication. However only 12.5% of the examined pharmacist gave a complete correct answers related to drug-herb interactions. Figure 4 illustrates these results.

**Fig. 5:**
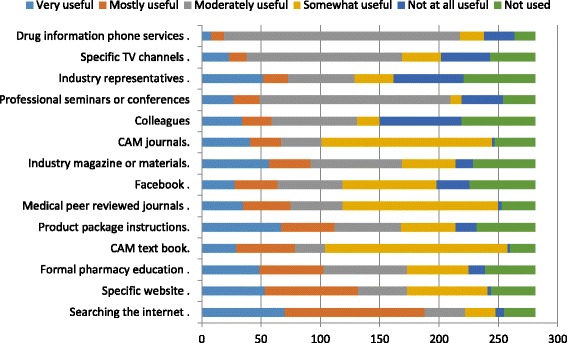
Information resources used by pharmacists

With regard to the required information about CAM during the past year, our data showed that the pharmacists sought information about CAM such that drugs interactions 200 (71.2%) and CAM use in pregnancy 199 (70.8%) were the most common needed information. Table 7 summarizes our findings. Pharmacists were asked to identify sources of information they find to be useful when they look up for information about CAM. As shown in Fig. 5, searching the internet 118 (42.0%) was reported as the most useful source for seeking information, whereas drug information phone services was not used very often by the pharmacists 199 (70.8%).

**Table 7:** Types of information needed in order to make appropriate recommendations by the pharmacists about CAM

Information	Yes No.(%)
Drug interaction	200 (71.2)
Use in pregnancy	199 (70.8)
Side effects	182 (64.8)
Efficacy is evidence based	179 (63.7)
Cautions of use	177 (63)
Use in children	168 (59.8)
Dose	129 (45.9)
Use	102 (36.3)
Patient information	88 (31.3)
Choice of the product	69 (24.6)
Supplier	45 (16.0)

## Discussion

Palestine has a unique geographical location at the meeting point of the three continents (Asia, Africa and Europe). This location plays an important role in diversity of CAM products and encourages people here to use them. Community pharmacists, as an important part of the health care system, are in a position to give information to the customers with evidence-based information [38]. Hence, to our best of knowledge, this study is the first survey in Palestine that investigate the community pharmacists practice, perception, beliefs about CAM and knowledge about different types and modalities of CAM in general and not only herbal medicines.

### Demographic characteristics of the participant pharmacists

The socio-demographic characteristics of pharmacists in Palestine are similar to those in other parts of the world being mainly males (53.0%) [29, 30, 38]. Contrary to other published data, in which females were the majority of the pharmacists [6, 33]. Our data showed that the age of participant pharmacists ranged from 20 to 72 years (age was 35 ± 1.08 years; mean ± SD). The predominant age group being between 20 and 29 years (39.9%).Compared to the study conducted Jordan, the results regarding the range of age and years of experience of pharmacists were almost similar [29]. However, Kheir and his coauthors in Qatar found that the average age was higher, therefore they had higher years of experience [6].

Regarding pharmacy practice pertaining to CAM products, different types of CAMs were recommended by the Palestinian pharmacists. Exercises and food supplements were the two major recommended types. These results are almost in line with the findings of Awad (2012) in Kuwait where the most commonly used CAM modalities among students were herbal products, massage, nutritional supplements [33]. Similarly, in Iranian survey, herbal therapy, cupping and massage therapy were considered of medium or high level efficiency by more than 60% of participants [39]. This in part is consistent with the Kuwait study but somehow different from the USA study in which the most frequently used alternatives to conventional medicine were relaxation techniques, chiropractic, and massage [33, 40, 41].

However, ritual Islamic spirituality including Quran, fasting, prayer, hejama and zamzam had a reasonable percent of pharmacist’s recommendations. The popularity of these types of CAM comes from its religious value and cultural believes. Alfaris et al. [42] have found prayer and spiritual healing as one of the most often used complementary and alternative therapy in Saudi Arabia. Since the highest percent of responders were Muslims with the same believes as Saudi people, that’s why the recommendations of ritual Islamic spirituality were reasonable.

Pharmacists were also asked whether they prescribe supplements and herbs during the last year. Vitamin B_12_ (93.2%), fish oil (86.8%), multivitamins (85.4%) were the highest prescribed agents. Deficiency of vitaminB_12_is prevalent during all age groups was reported in different developing countries. Inadequate intake, due to low consumption of animal-source foods is the main cause of low serum vitamin B_12_ [43]. In Palestine, it was noted that during the last several years, serum vitamin B_12_ determination was more frequently requested by the physicians and vitamin B_12_ deficiency becomes as if it is an epidemic disease [44].

### Pharmacists’ practice and beliefs about CAM

Our results showed that the leading factor affecting pharmacist’s recommendation of specific CAM product was the proved efficacy of the product (87.2%) followed by the customer’s positive responses regarding product effectiveness (77.6%). Australian study conducted by Bushett and coauthors [12] resulted in that brand familiarity was the main influence in pharmacist decision although the majority of pharmacists (88.2%) recommends generic name of the product only. Regarding the beliefs of pharmacists about CAM, the majority of the participants (96.5%) in this study agreed that CAM need more scientific testing before use. This finding is consistent with the results of a previous survey conducted by Sweileh et al. [37], in which only 31% of respondents agreed that herbal drugs have been sufficiently studied. In the Australian study the result was also very close to our result, in which (91%) of the pharmacists agreed that CAMs need more scientific testing [34]. Clearly, these results along with results of other studies show a global agreement among pharmacist on the need of CAM to be scientifically evident [6, 33, 45].Interestingly, these results negates the general perception of CAM being ‘safe ‘[45].

A fair number of pharmacists (42%) have a positive beliefs about CAM regarding the statement that results of CAM are mainly due to placebo effect. While, in the Australian study majority of the pharmacist have a neutral attitude about this statement (44%) [34]. This can be attributed to cultural background and strong belief in benefits of CAM products among Palestinian pharmacists in the recent study, generally, people’s cultural and ethnic backgrounds can influence their propensity for using CAM [46].

When comparing CAM with conventional medicine, non-surprisingly, only (27.1%) of the participants agreed with the statement that CAMs can offer therapeutic benefits that the conventional medicine does not, whereas (44.1%) disagreed. To some acceptable extent, this result agrees with a Singaporean study, which reported that only 15% of pharmacist recommended CAM because they are unsatisfied with results of conventional medicine, while 26% were satisfied with conventional medicine. This can reflect a general trend among pharmacists to CAMs as any complement medicine rather than alternative to conventional medicine, and dissatisfaction with conventional medicine is not necessarily the reason for turning to CAM.

Interestingly, the majority of the pharmacists (64.4%) agreed that CAM improve general health and not only cure the disease, in agreement with the Singaporean study, the main reason for using CAM was reported as improving general health and promote wellbeing [33]. This supports the idea of CAM as a preventive as well as curative measure to treat health problems. A great majority of the pharmacists (76.5%) showed agreement with statement that pharmacists should regularly ask consumer if they use CAM, positively, this reflects the awareness of pharmacist upon safety measurements and drug-herbal interactions that can affect consumer’s safety. In the Australian study, surprisingly, the percent was 100, which reflects a high level of awareness. Eventually, research regarding both conventional and CAM therapies is ongoing and the medical evidence can change rapidly, the clinician should communicate regularly with the patient regarding any new developments [47].

When comparing the beneficial effect of CAMs with conventional medicine, we asked the respondents weather they agree that alternative medicine offers the patient benefits not offered by conventional medicine. Only 27.1% agreed with this statement while in an Australian study about Complementary Medicines among hospital pharmacists, conducted by Brown (2009), the results were close to ours in which only 35% agreed with this statement. Our result is much lesser than the result of Sweileh et al. [48] study which reported that 61% of pharmacists believed that herbal medicine was more beneficial and safer than conventional medicine. This could be attributed to either the time/regional difference between the two studies or the fact that Sweileh’s study was focused only in herbs and therefore the numbers were higher when compared to our results which focused on CAM in general.

In this study more than half of the pharmacists 57.3% (161 pharmacists) showed confidant discussing CAM with the consumers. Comparing this with their knowledge score (a score of 4.32 ± 1.78, mean ± SD), the level of confidence is conformed to their fair knowledge. However, in the previous survey by Sweileh, majority of pharmacists rated their knowledge as good while the actual testing scores, which was reported to be poor, did not substantiate this impression [37]. On the other hand, the Australian study investigating hospital pharmacist attitude, reported lack of both confidence and knowledge. Generally, pharmacists showed fair beliefs about CAM, based on beliefs about CAM score test with a mean of 4.2 of 7.

Besides, several approaches were made to correlate pharmacist attitude with demographic characteristics, as shown in Table 3, there was no significant difference in beliefs score within the socio-demographic groups. Regarding the barriers that limit appropriate CAM recommendation by the pharmacists, the most reported two in this study are the small number of well-trained pharmacists on CAM use (82.6%) followed by lack of scientific knowledge in CAM (73.7%).

### Pharmacists’ knowledge about CAM

The finding of this research draw an attention on patient safety and pharmacists educational and training needs. The pharmacists’ scores from the knowledge test were fair, with a median of 5.00 ± 1.78 (mean score 4.32 ± 1.78) out of 8 questions. Pharmacists were more likely to answer statements about the side effect and contraindication of the herbal products as shown in Fig. 1, this sounds good regarding patient safety, which is one of the biggest concerns related to CAM use. However, since the percentage of responders to these questions was low (24.2%), we should not neglect the importance of increasing pharmacists awareness of the potential harmful effects that CAM may cause by Continuing Education and Training.

The observed knowledge score is higher than a previous survey about pharmacists knowledge of natural herbal product in Qatar where pharmacists mean score was 4.51 ± 3.57 (mean ± SD), when they answered 12 questions about knowledge of natural herbal product [6]. Inappropriately, our study illustrated that Palestinian pharmacists’ knowledge scores are lower than the survey done in Singapore about pharmacists knowledge toward CAM with a mean score of 7.23 ± 1.96 (mean ± SD), when pharmacists answered the knowledge test consisting of ten questions [45].

No differences were found in knowledge score between males and females, whereas in Qatar survey female pharmacists showed superior knowledge scores than males [6]. However, there is a difference found in knowledge score between the different groups of age and years of experience. With the lower age, lower years of experience having the highest knowledge score. A possible explanation for this might be that the newly graduated pharmacists had taken relevant courses about CAM during their undergraduate studies. These results related to the years of experience conform to the result of survey published in 2013 about Dispensing Practices, Attitudes, and Knowledge of Pharmacists towards Herbal Products in Palestine [37].

As expected, there is statistically significant difference in knowledge score based on educational level. Pharmacists with postgraduate studies (master and PhD degree) have a higher knowledge score, this is logical due to the increased years of education with more courses being studied that may be related somehow to CAM.

One interesting finding is the significant difference between knowledge score and the University of Graduation, with the pharmacists locally graduated from Palestinian universities having the highest knowledge score. This may reflect the improved educational levels offered by local universities and the attention given to the common CAM in our countries, by offering courses about their indications, side effect, contraindications and the interactions. The pharmacists knowledge scores are higher in those where their pharmacies located in village, this may be due to the wide believe in CAM in the village, where the community pharmacists are the first health care provider from whom patients seek recommendations on CAM products.

The increasing number of patients using CAM pose a challenge to pharmacists. Therefore, pharmacists need to incorporate CAM learning into their continuing professional development plan to improve their knowledge and skills in dealing with patients taking CAM. Educational meetings alone or combined with other interventions, can improve professional practice and healthcare outcomes for the patients [49].

Echinacea is one of the available herbal products found in our pharmacies. Echinacea preparations, commonly perceived as herbal immune stimulants or “cold fighters,” are among the most widely used dietary supplements in Europe and the United States [50]. As the results show only (33.8%) were familiar with its indication, while (91%) of community pharmacists in Riyadh, Saudi Arabia were more familiar with this indication [31].

Finally, pharmacists were least familiar with the drug – herb interactions (12.5%). Only about (19.6%) were aware of the interaction of digoxin with bran. Whereas, more than half of the pharmacists were aware of Garlic (*Allium sativum*) and warfarin interaction in term of bleeding. As shown in the practice part Garlic (*Allium sativum*) is prescribed by (42.7%) of the pharmacists. In 2007, a published research about the effects of garlic on platelet biochemistry and physiology concluded that garlic inhibits platelet aggregation by multiple mechanisms and may have a role in preventing cardiovascular disease [51]. Another study showed that CAM use in Asian patients is prevalent and associated with the ‘chronic disease triad’ (of arthritis, musculoskeletal disorders and stroke), satisfaction with care and cultural beliefs [52]. This put an effort on the pharmacists to be more aware of this interaction.

### Information seeking behaviors

The most frequently needed information are related to the safety of CAMs, which included drug interactions (71.2%), use in pregnancy (70.8%) and side effects (64.8%). These results are in agreement with those obtained by Bushett and coauthors [12] in Australia, in which the percentage of pharmacists needed the above information were 95% for drug interactions, 76% for contraindications and 75% for side effects. It was expected that the most information needed among participant is about CAM types and drug interactions as Sawalha’s [22] study in Palestine showed that there was a significant correlation (p -value of 0.039) between the number of health conditions treated and the number of CAM types used. Also in the same study, herbal therapy was the most commonly used. Pokladnikova et al. [53] showed that pharmacists obtained information on CAM mainly from pharmacy journals (69%) and the internet (60%) [53]. In our study, the main source of information was internet (66.9%) and specific websites (47%). It is not surprisingly that internet research has the highest percentage, because there is a wild spread of the modern mobile phones with many applications that help to reach to the information faster and easier.

## Limitations

There were some limitations in the current study. Firstly, the method involved a self-administered questionnaire, so response bias is likely. The use of self-evaluation and limited questions for evaluation of knowledge are limitation of this research. Normally, the best way for practice evaluation is achieved through observation. The generalization of the results is limited as the sample of pharmacists was taken from West Bank alone which may not be representative of all Palestinian pharmacists. In addition, participant pharmacists were asked about CAM dispensed in the last year, which is prone to recall bias, as they may find it difficult to remember exactly what products were used. Finally, using internet as a source of seeking information is broad, there are reputable sites and non-reputable sites on the internet and it could be hard to deduce the quality of information obtained from different web sites.

## Conclusions

In conclusion, this study showed that more than 76% of participant agreed that the pharmacist must constantly inquire if the patient is using any type of CAM but rate their own knowledge about CAMs as inadequate, and were not fully confident in answering patient’s questions. The majority of pharmacists reported that the two main factors affected their recommendations of a specific product are the effectiveness and the scientific approval of the product. CAM recommendations by pharmacists appear to be commonplace. Although their knowledge scores were fair to average, pharmacists still need more education and training about CAM in order to be more qualified to provide better pharmaceutical care and improve their patient’s outcome. This might necessitate that regulatory bodies should pay more attention to CAM resources at this setting, including herbal medicines for pharmacy students as part of the undergraduate curriculum and more structured training schedule for practicing pharmacists.

## Abbreviations

CAM: Complementary and alternative medicine
IRB: Institutional review board
PMS: Premenstrual syndrome
SD: Standard deviation
SPSS: Statistical package for social sciences
